# A primary healthcare information intervention for communicating cardiovascular risk to patients with poorly controlled hypertension: The Education and Coronary Risk Evaluation (Educore) study—A pragmatic, cluster-randomized trial

**DOI:** 10.1371/journal.pone.0226398

**Published:** 2020-01-23

**Authors:** Esperanza Escortell-Mayor, Isabel del Cura-González, Elena Ojeda-Ruiz, Teresa Sanz-Cuesta, Isidro Rodríguez-Salceda, Jesús García-Soltero, María-José Rojas-Giraldo, Pedro Herrera-Municio, Alicia Jorge-Formariz, Ángela Lorenzo-Lobato, Luisa Cabello-Ballesteros, Rosario Riesgo-Fuertes, Sofía Garrido-Elustondo, Mariel Morey-Montalvo, Milagros Rico-Blázquez, Ricardo Rodríguez-Barrientos, María-Dolores Fuente-Arriaran, Gloria Sierra-Ocaña, Encarnación Serrano-Serrano, Carmelina Sanz-Velasco, Roberto Carrascoso-Calvo, Juan Carlos Recio-Velasco, Marta Sanz-Sanz, Mercedes Rumayor-Zarzuelo, Olga-Inés Bermejo-Mayoral, Josefina Galán-Esteban, Antonio Sarría-Santamera, Educore Group

**Affiliations:** 1 Unidad de Apoyo a la Investigación, Gerencia Asistencial de Atención Primaria, Madrid, Spain; 2 Red de Investigación en Servicios de Salud en Enfermedades Crónicas (REDISSEC), Madrid, Spain; 3 Área Medicina Preventiva y Salud Pública, Departamento Especialidades Médicas y Salud Pública, Facultad de Ciencias de la Salud, Universidad Rey Juan Carlos, Alcorcón, Madrid, Spain; 4 Centro Nacional de Epidemiología, Instituto de Salud Carlos III (ISCIII), Madrid, Spain; 5 Centro de Salud La Veredilla, Torrejón de Ardoz, Madrid, Spain; 6 Centro de Salud Santa Isabel, Madrid, Spain; 7 Centro de Salud Parque Loranca, Fuenlabrada, Madrid, Spain; 8 Centro de Salud Alcalá de Guadaira, Madrid, Spain; 9 Unidad Docente Multiprofesional de Atención Familiar y Comunitaria Noroeste, Madrid, Spain; 10 Unidad Docente Multiprofesional de Atención Familiar y Comunitaria Sur. Getafe, Madrid, Spain; 11 Unidad Docente Multiprofesional de Atención Familiar y Comunitaria Sureste, Madrid, Spain; 12 Servicio de Prevención de la Enfermedad, Subdirección General de Promoción, Prevención y Educación para la Salud, Dirección General de Salud Pública, Madrid, Spain; 13 Centro de Salud Sector 3, Getafe, Madrid, Spain; 14 Centro de Salud Manuel Merino, Alcalá de Henares, Madrid, Spain; 15 Centro de Salud Los Fresnos, Torrejón de Ardoz, Madrid, Spain; 16 Centro de Salud Andrés Mellado, Madrid, Spain; 17 Centro de Salud Las Águilas, Madrid, Spain; 18 Área Única de Salud Pública-2, Dirección General de Salud Pública, Madrid, Spain; 19 Departamento de Gestión de Servicios Sanitarios, Escuela Nacional de Sanidad, Instituto de Salud Carlos III (ISCIII), Madrid, Spain; 20 Facultad de Medicina y Farmacia, Universidad de Alcalá, Alcalá de Henares, Madrid, Spain; Kurume University School of Medicine, JAPAN

## Abstract

**Purpose:**

Uncertainty exists regarding the best way to communicate cardiovascular risk (CVR) to patients, and it is unclear whether the comprehension and perception of CVR varies according to the format used. The aim of the present work was to determine whether a strategy designed for communicating CVR information to patients with poorly controlled high blood pressure (HBP), but with no background of cardiovascular disease, was more effective than usual care in the control of blood pressure (BP) over the course of a year.

**Methods:**

A pragmatic, two-arm, cluster-randomized controlled trial was performed. Consecutive patients aged 40–65 years, all diagnosed with HBP in the last 12 months, and all of whom showed poor control of their condition (systolic BP ≥140 mmHg and/or diastolic BP ≥90 mmHg), were recruited at 22 primary healthcare centres. Eleven centres were randomly assigned to the usual care arm, and 11 to the informative intervention arm (Educore arm). At the start of the study, the Educore arm subjects were shown the "low risk SCORE table", along with impacting images and information pamphlets encouraging the maintenance of good cardiovascular health. The main outcome variable measured was the control of HBP; the secondary outcome variables were SCORE table score, total plasma cholesterol concentration, use of tobacco, adherence to prescribed treatment, and quality of life.

**Results:**

The study participants were 411 patients (185 in the Educore arm and 226 in the usual care arm). Multilevel logistic regression showed that, at 12 months, the Educore intervention achieved better control of HBP (OR = 1.57; 1.02 to 2.41). No statistically significant differences were seen between the two arms at 12 months with respect to the secondary outcomes.

**Conclusions:**

Compared to usual care, the Educore intervention was associated with better control of HBP after adjusting for age, baseline SBP and plasma cholesterol, at 12 months.

## Introduction

Most Spanish patients with high blood pressure (HBP) are attended to at primary healthcare centres in the Spanish National Health System. This easy-access first level of assistance provides integrated and continuous care [1–3]. But, it is complex to provide appropriate information to patients in the healthcare environment.

HBP control figures might be improved via the use of effective pharmacological [4,5] and non-pharmacological intervention strategies [6,7]. According to some authors, a major challenge of such interventions is getting the patient on board. Certainly, family doctors need to transmit information on the risks and benefits of treatment alternatives in a rigorous yet understandable fashion [8]. One randomized clinical trial [9] reported the skill of patients in identifying cardiovascular risk (CVR) factors to improve when communication was made personal. Other authors [10] have reported that medical professionals normally communicate these risks verbally, using words and or figures; they also indicate, however, that controversy exists regarding the best way to communicate with patients, particularly over whether verbal communication is improved with visual aids.

The effectiveness of different interventions for communicating CVR information and the impact on risk comprehension and intention to modify personal behaviour has been compared in a systematic review [11]. The authors concluded that more clinical trials were needed to determine how graphic aids used in explanations affect the understanding of risk. In one such trial [12], patients were shown information in different graphic formats and their taking of treatment decisions assessed. It was concluded that pictograms might be the best format, especially when dealing with patients with weak numerical skills.

The *Education and Coronary Risk Evaluation* (EDUCORE) study aims to examine the importance of using visual information for instructing patients about CVR and its effect on HBP control. The present work compares, in the primary healthcare setting, usual care and a CVR communication strategy with respect to the achievement of good control of HBP in patients in whom this was poorly controlled but who had no cardiovascular disease (CVD).

## Methods

### Design

This work was designed as a pragmatic, two-arm, cluster-randomized controlled trial lasting one year. It was performed in the primary healthcare setting. The health centres (clusters) were the randomized units, and the patients the analysis units. The supporting CONSORT checklist is available as supporting information; see S1 File.

The study was approved by the Ethics Committee of the *Hospital Universitario Príncipe de Asturias* (2009/24/06) and was registered at ClinicalTrials.gov (NCT01155973) Specifics of the methodology followed can be found in the study protocol [13]; see S2 File. There were no deviations from this study protocol.

Twenty-two health centres in eight municipalities representative of the Madrid Region (Madrid City, Torrejón de Ardoz, Alcalá de Henares, Alcobendas, Fuenlabrada, Leganés, Pinto and Getafe) were selected on the basis of their being teaching centres and/or their having participated before in research studies. All the health professionals involved in the study at these centres volunteered their efforts.

### Study population

The study patients were aged 40–65 years; all were recruited between June 29, 2010, and August 11, 2011 and completed the follow-up one year later.J All had received a diagnosis of HBP in the previous 12 months, and in all it was poorly controlled (systolic blood pressure (SBP) ≥140 mmHg and/or diastolic blood pressure (DBP) ≥90 mmHg; these criteria have remained unchanged across Europe for some years [5]). Patients with CVD and diabetes mellitus were excluded.

### Sample size

The sample size was determined contemplating a 15% greater increase in the number of patients showing good HBP control in the intervention arm than in the usual care arm, and α value of 0.05, and for a power of 80%. According to data from Spanish studies, good control of SBP and DBP is around 41.4% of patients. The minimum sample size was corrected for the design effect contemplating an intraclass correlation coefficient of 0.03 [14], and assuming a mean cluster size of 30 patients. The final sample size required was 736 patients, assuming 10% loss to follow-up over the study period.

### Randomisation

The 22 health centres (clusters) were assigned by simple randomization to the usual care arm or the information intervention arm—the Educore arm—by an independent statistician using Epidat 3.1 software (n = 11 in each arm). The health professionals at the centres then recruited patients in a consecutive fashion and doctors requested their informed written consent to participate. The same health professionals collected data on their age, sex, and professional category. The mean total number of patients seen per day at each health centre over 2011 was also recorded. These data were used to describe health centres’ characteristics.

### Intervention

**Fig 1:**
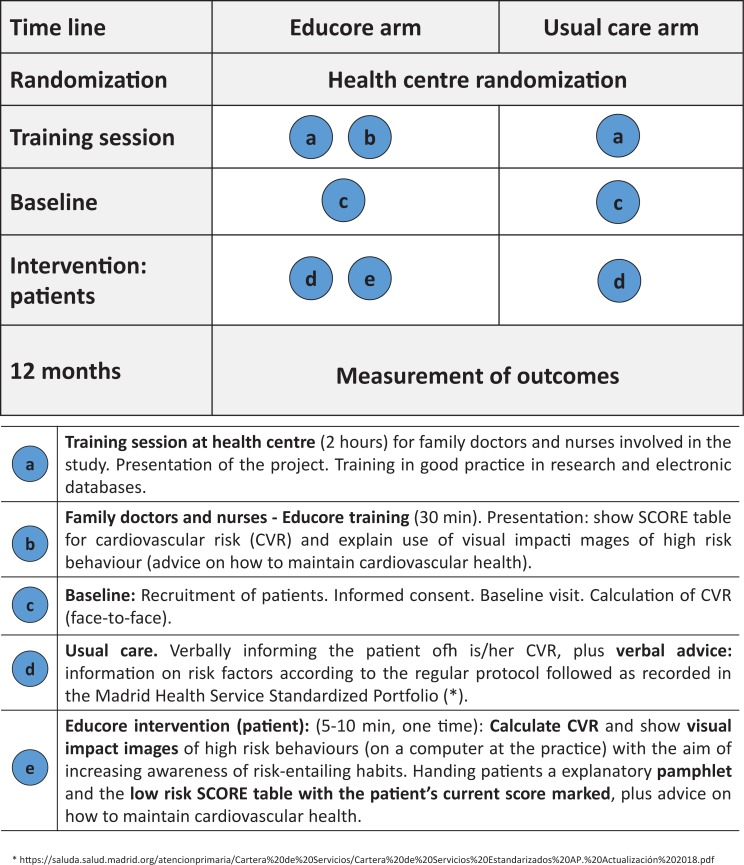
Graphical depiction of Educore intervention vs usual care.

The Educore intervention is complex in its nature [15]. It is directed towards patients with poorly controlled HBP and involves the visualization of risk via the "low risk Systematic Coronary Risk Evaluation (SCORE) table" [16], and through impacting images highlighting CVR. Information pamphlets were also provided that contained their SCORE table score, along with advice for maintaining good cardiovascular health. “Fig 1” describes both arms of the study in the manner currently recommended [17].

### Variables

The main outcome variable was good control of HBP (deemed to be good when SBP was <140 mmHg and DBP <90 mmHg). Two blood pressure (BP) readings separated by 2 minutes were taken at sitting position after a minimal resting of 5 minutes using a manually calibrated sphygmomanometer (following the clinical practice guidelines in Servicio Madrileño de Salud). If the readings were very different additional measurements were taken. For the trial an average value of the systolic and diastolic blood pressure was calculated. The secondary outcome variables were systolic BP (SBP) and diastolic BP (DBP), absolute SCORE table score, the plasma cholesterol concentration, use of tobacco, and compliance with treatment (as determined by the Morisky-Green questionnaire [18]), and mean quality of life as measured by the MINICHAL questionnaire [19,20]. The sociodemographic variables recorded were sex, age, level of education and physical activity undertaken. The anthropometric/clinical variables recorded were body mass index, consumption of antihypertension and lipid-lowering drugs, and any changes in treatment (with their motives).

Patient personal data were collected during clinical interviews lasting 10–30 min in the participating health professionals' offices. Results were recorded in an electronic data storage notebook at 0, 6 and 12 months—except SBP and DBP which were recorded at 0 (baseline), 3, 6, 9 and 12 months. The period of follow-up ended in December of 2012.

### Analysis

A descriptive analysis (means, medians, frequencies of distribution) was made of the healthcare centres and of the characteristics of the patients in each study arm.

The results for the primary outcome variable were analysed for intention to treat (ITT).

Missing values for the main outcome variable were added using the 'last observation carried forward' (LOCF) method [21]. Per protocol (PP) data analysis was also performed for the patients remaining in the study at 12 months.

Multilevel logistic regression analysis was performed to examine the control of HBP achieved in each arm. The dependent variable was good/bad control of HBP at 12 months, and the independent variable the intervention group to which each patient belonged. Additionally, the analysis included the covariates gender, age, and clinically important variables (baseline DBP, use of tobacco, body mass index, cholesterol level, and current antihypertensive treatment). Multilevel models are particularly appropriate when individuals cluster within groups and these groups share characteristics. To take into account this hierarchical structure, random effect terms are introduced to allow the effect of the different levels (in this case the health centres) to be estimated. Fixed effect variables can also be included. The random effect was quantified via the median OR (MOR) between centres, interpreted as the expected change (in medians) in good control of HBP for a patient who switches from one health center to another with increased risk.

Models were estimated using adaptive Gaussian quadrature with seven quadrature points per level. The likelihood ratio test was used to compare the models and assesses the goodness of fit [22].

The effect of the Educore intervention on the secondary outcome variables (SBP/DBP, CVR, cholesterol levels, use of tobacco, compliance with treatment, and quality of life) were determined at 12 months using appropriate multilevel statistical tests for comparation between arms (multilevel mixed-effects linear regression and multilevel mixed-effects logistic regression).

Significance was set at p<0.05. All calculations were made using SPSS 21 and STATA 14 software.

## Results

**Fig 2:**
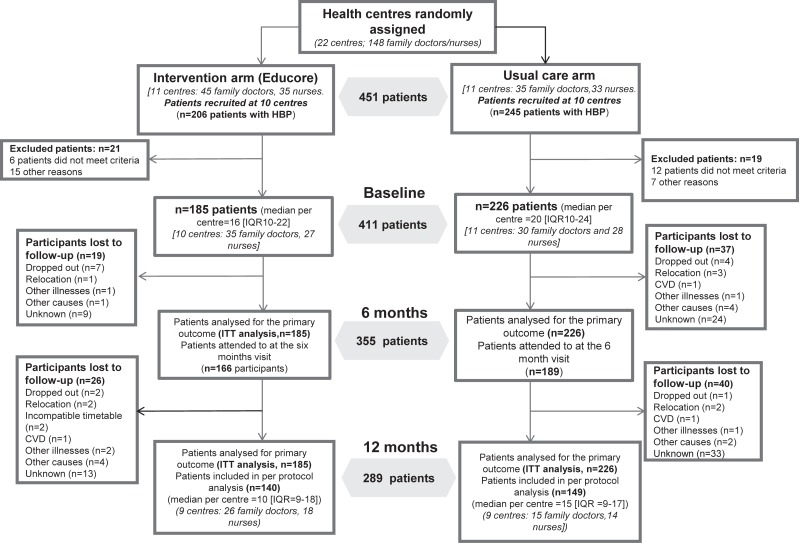
Study flow diagram.

The final study sample included 411 patients, 226 in the usual care arm and 185 in the Educore arm. At 6 months, 86% of the patients remained in the study, falling to 70% at 12 months (289 patients). The median number of patients recruited per health centre was 16 for the Educore arm and 20 for the usual care arm. “Fig 2” shows the flow diagram for the patients entering each arm, and those remaining at each data collection point, according to the recommendations for presentations made by the CONSORT-Cluster Group [23].

The number of participating health professionals at the 21 health centres was 120 (65 family doctors and 55 nurses) “Table 1”.

**Table 1:** Baseline characteristics of the health centres (clusters) and patients in the usual care and Educore arms.

Characteristics of health centres#		Educore arm	Usual care arm	TOTAL
***Health centres (n = 21)***		**(n = 10)**	**(n = 11)**	**(n = 21)**
**Daily work of family doctors (patients/day)**	Mean (SD)	33 (2.5)	34 (3.1)	33 (2.9)
**Daily work of nurses (patients/day)**	Mean (SD)	19 (1.2)	19 (3.1)	18.8 (2.3)
***Health professional characteristics (n = 120)***		**(n = 62)**	**(n = 58)**	**(n = 120)**
**Age (years)**	Mean (SD)	46.2 (6.6)	45.9 (8.3)	46 (7.5)
**Female sex**	(%)	83.6	69.1	76.4
**Family doctors**	(%)	56.4	43.6	53.6
**Nurses**	(%)	50.9	49.1	46.4
**Characteristics of patients**		**Educore arm (n = 185)**	**Usual care arm (n = 226)**	**TOTAL (n = 411)**
**Female sex (n = 411)**	(%)	46.5	55.8	51.6
**Age (years) (n = 411)**	Mean (SD)	55.5 (7)	55.2 (6.5)	55.3 (6.7)
**Education level (n = 407)**				
- No degree	(%)	61.4	60.1	60.7
- Bachelor’s degree or above	(%)	38.6	39.9	39.3
**Physical activity** (≥3h/week) **(n = 411)**	(%)	57.8	62.8	60.6
**METs (n = 411)**	Mean (SD)	10.0 (12.7)	11.1 (15.0)	10.6 (14.0)
**Use of tobacco (n = 411)**				
- Never smoked	(%)	48.1	54.0	51.3
- Ex-smoker	(%)	25.4	22.1	23.6
- Smoker	(%)	26.5	23.9	25.1
**No of cigarettes/day (n = 102)**	Mean (SD)	15.7 (9)	15.5 (10.0)	15.6 (9.4)
**Packs/year (n = 102)**	Mean (SD)	24.1 (16.5)	23.8 (16.6)	24 (16.5)
**BMI (kg/m**^**2**^**) (n = 408)**	Mean(SD)	31.0 (5.9)	30.7 (5.5)	30.8 (5.1)
- <25 kg/m2	(%)	10.4	13.8	12.3
- 25–29 kg/m2	(%)	36.1	32.9	34.3
- ≥30 kg/m2	(%)	53.6	53.3	53.4
**Systolic blood pressure (mm Hg) (n = 411)**	Mean (SD)	147.0 (10.7)	147.7 (11.6)	147.4 (11.2)
**Diastolic blood pressure (mm Hg) (n = 411)**	Mean (SD)	90.7 (8.6)	89 (8.3)	89.8 (8.5)
**Cholesterol (mg/dl) (n = 398)**	Mean (SD)	215.9 (32.4)	212.6 (32.0)	214.1 (32.2)
**LDL-cholesterol (mg/dl) (n = 372)**	Mean (SD)	137.7 (32.7)	131.9 (30.1)	134.5 (31.4)
**SCORE table score for CVR (n = 396)**	Mean (SD)	2.5 (2.4)	2.5 (3.1)	2.5 (2.8)
- Low risk (<1%)	(%)	11.7	12.9	12.4
- Moderate risk (1–4%)	(%)	71.5	72.8	72.2
- High risk (5–9%)	(%)	15.1	12.4	13.6
- Very high risk (≥10%)	(%)	1.7	1.8	1.8
**Mini-CHAL Test (n = 410)**				
- Mood	Mean (SD)	4.7 (4.1)	4.3 (3.9)	4.5 (4.0)
- Somatic status	Mean (SD)	2.4 (2.4)	2.0 (2.3)	2.2 (2.3)
**Compliance with treatment (n = 411)**	(% Yes)	71.9	72.1	72.0
**Number of medications (n = 411)**	Mean (SD)	1.3 (0.9)	1.4 (0.9)	1.36 (0.9)
**Medication changes (n = 411)**	(%)	43.2	31.2	36.9
**Antihypertensive agents (n = 411)**	(%)	78.9	75.2	76.9
**Monotherapy**	(%)	39.5	48.2	44.3
- Angiotensin-converting enzyme inhibitors	(%)	40.5	43.8	42.3
- Diuretics	(%)	39.5	39.4	39.4
- Angiotensin II Type 1 receptor blockers	(%)	22.2	20.8	21.4
- Calcium channel blockers	(%)	16.2	17.3	16.8
- Adrenergic beta-antagonists	(%)	18.4	9.7	13.6
- Adrenergic alpha-antagonists	(%)	3.2	1.3	2.2
**Lipid-lowering drugs (n = 411)**	(%)	19.5	18.1	18.7

# **Source:** *Sistemas de Información*. *Gerencia Asistencial de Atención Primaria*. **METs** (Metabolic equivalent hours/week). **Mini-CHAL Test:** Zero (best health level) to 30 (worst health level) for mood, and 0 to 18 for somatic problems.

The mean age of the patients was 55.3±6.7 years; 51.6% were women. At the start of the study, 15.4% of the patients were SCORE classified as being at high or very high risk; 67.3% had high plasma cholesterol (mean 214.1 mg/dl), and 25.1% declared themselves smokers. With respect to quality of life, the mean mood score was 4.5 (on a scale of 0–30 from better to worse), and the mean somatic manifestation score was 2.2 (on a scale 1–18 from better to worse). 23.1% of the patients initially took no antihypertension medication; 44% were on monotherapy, and 40% took two or more medications.

The most commonly prescribed medications were angiotensin-converting-enzyme inhibitors (ACE inhibitor), diuretics and angiotensin II receptor blockers (ARBs). Under 15% of the patients took beta-blockers. 39.7% patients received more than one class of antihypertensive medication and 11.2% received more than two. No statistical significance difference were found between groups. The most common combined treatment found was ACE inhibitor+ Diuretics (11.7%). Almost 20% had been prescribed lipid-lowering drugs. Some 70% of patients declared their adherence to pharmacological treatment. “Table 1” shows the baseline values for the cluster, medical personnel and patient variables for both arms of the trial.

### Primary outcome

The control of HBP improved in both arms at 6 and 12 months (55.7% and 67.6% respectively in the Educore arm [p<0.001 between these two time points], and 50.4% and 58.8% respectively in the control arm [p<0.001 between these two time points]; ITT analysis). “Table 2” shows the results for the control of HBP for both the per protocol and ITT analyses at 6 and 12 months; without adjustment for any factor, the improvements observed do not differ significantly. However, after adjusting for age, baseline SBP and plasma cholesterol, the Educore intervention was associated with better control of HBP at 12 months (OR 1.57; 95%CI (1.02–2.41)). The MOR (median odds ratio) between centres was 1.14, which can be interpreted as the median odds ratio of good control of HBP across all patients in different health centres (comparing the higher risk to lower risk centres). The MOR of 1.14 is lower than the intervention OR, suggesting that variation between health centres contributed less to good control of HBP than did the intervention. “Table 3”.

**Table 2:** Good control of high blood pressure at 6 and 12 months (per protocol and intention to treat analyses).

EndpointGood control	Educore arm		Usual care arm		Unadjusted OR(95%CI)	Adjusted OR by cluster (95%CI)	Adjusted OR by cluster and others covariables‡(95%CI)
	n	% control	n	% control			
**6 months**							
PP	166	59.0%	189	54.5%	1.203 (0.773; 1.876)	1.254 (0.668; 2.354)	1.302 (0.712; 2.381)
ITT	185	55.7%	226	50.4%	1.234 (0.819; 1.858)	1.326 (0.723; 2.43)	1.375 (0.778;2.429)
**12 months**							
PP	140	74.3%	149	66.4%	1.459 (0.851; 2.512)	1.457 (0.869; 2.443)	1.451 (0.858; 2.454)
ITT	185	67.6%	226	58.8%	1.457 (0.952; 2.235)	1.487 (0.870; 2.542)	1.575 (1.022; 2.426)

OR: Odds ratio. Per Protocol [PP] analyses. Intention To Treat [ITT] analyses

‡ Age, sex, baseline Systolic blood pressure basal, baseline Cholesterol, number of **antihypertensive agents**

**Table 3:** Good control of high blood pressure at 12 months according to multilevel logistic regression analysis.

Variables	OR	95%CI
**Arm**‡	1.568	(1.019; 2.413)
**Age**	1.039	(1.005; 1.073)
Baseline systolic blood pressure	0.959	(0.941; 0.979)
Baseline cholesterol	0.992	(0.985; 0.999)
number of antihypertensive agents	0.947	(0.749; 1.198)

‡ Reference category: usual care arm. Intention to treat analysis (n = 398).

OR: Odds ratio. Random effect variable: health centre (var/MOR) 0.164/1.141). p<0.001 for random effect variables (Health Centre): var, variance; MOR, median OR.

### Secondary outcomes

In both arms, significant reductions in SBP and DBP were achieved over time “Table 4”, but with no significant difference between the arms.

**Table 4:** Changes in systolic and diastolic blood pressure over time, within and between the Educore and usual care arms.

	EDUCORE cluster (n = 185) ‡			Usual care cluster (n = 226) ‡			Unadjusted difference between groups
	mean (SD)	Difference in means‡‡‡(95% CI)	SRM	mean (SD)	Difference in means ‡‡ (95% CI)	SRM	
**SBP (mmHg**)							
**Baseline**	147.04 (10.73)			147.71 (11.62)			
**3 months**	133.86 (12.52)	-13.18 (-15.07; -11.29)	-1.012	137.25 (15.15)	-10.46 (-12.52; -8.40)	-0.667	-3.39 (-7.45; +0.67)
**6 months**	133.57 (12.85)	-13.48 (-15.51; -11.44)	-0.962	135.02 (15.11)	-12.70 (-14.73; -10.66)	-0.818	-1.45 (-4.46; +1.56)
**9 months**	132.40 (13.05)	-14.64 (-16.68; -12.61)	-1.043	134.00 (14.50)	-13.72 (-15.73; -11.70)	-0.891	-1.60 (-4.50; +1.31)
**12 months**	131.30 (12.52)	-15.74 (-17.78; -13.71)	-1.122	133.08 (14.79)	-14.64 (-16.61; -12.66)	-0.971	-1.77 (-4.87; +1.32)
**DBP (mmHg)**							
**Baseline**	90.69 (8.63)			89.0 (8.32)			
**3 months**	83.11 (8.62)	-7.57 (-8.87; -6.27)	-0.843	82.57 (9.80)	-6.43 (-7.82; -5.05)	-0.608	0.55 (-2.61; +3.70)
**6 months**	82.08 (8.99)	-8.61 (-10.08; -7.15)	-0.852	81.93 (9.30)	-7.07 (-8.40; -5.74)	-0.697	0.15 (-2.26; +2.56)
**9 months**	81.26 (8.42)	-9.42 (-10.92; -7.92)	-0.910	80.72 (9.17)	-8.28 (-9.58; -6.98)	-0.835	0.55 (-1.66; +2.75)
**12 months**	81.03 (8.47)	-9.66 (-11.09; -8.23)	-0.972	80.40 (9.55)	-8.60 (-9.97; -7.23)	-0.821	0.62 (-1.71; +2.96)

SBP: Systolic Blood Pressure; DBP: Diastolic Blood Pressure; SRM: Standardized Response of the Mean. A negative SRM denotes improvement; a positive one denotes the worsening of some clinical measurement.

‡ Missing data replaced using Last observation Carried Forward (LOCF).

‡‡ Compared to baseline.

The mean SCORE table score fell from a 2.5 in both arms (baseline) to 2.0 in the Educore arm and 2.1 in the usual care arm at 12 months. At 12 months, no patient was at very high CVR in either arm.

A positive intervention effects at 12 months, according to multilevel regression analysis, over all secondary outcomes except mental status del Mini-CHAL was found. However, these differences were not statistically significant. Table 5

**Table 5:** Intervention effects over secondary outcomes at 12 months according to multilevel regression analysis.

**Outcome measure (at 12 months)**	**Total**	**Educore (n = 140)**	**Usual care (n = 149)**	**Coefficient Adjusted by cluster and basal level (95% CI)**
**SCORE table score for CVR (n = 264)** ‡	2.03 (1.67)	1.98 (1.61)	2.08 (1.74)	-0.367(-0.905; 0.170)
**Cholesterol (mg/dl) (n = 281)** ‡	207.6(33.3)	206.5 (32.7)	208.6 (33.9)	-0.870 (-9.460; 7.718)
**Quality of life (Mini-CHAL)** ‡				
- Mental status **(n = 262)**	3.1 (3.5)	3.1 (3.4)	3.0 (4.0)	0.369 (-0.782;1.521)
- Somatic status **(n = 268)**	1.4 (2.2)	1.2 (2.0)	1.5 (2.3)	-0.372 (-0.882;1.388)
** **		**Educore** (n = 140)	**Usual care (n = 149)**	**OR Adjusted by cluster (95% CI)**
**Use of tobacco (Yes) (n = 289)**	20.5%	19.3%	21.6%	0.866 (0.487;-1.537)
**Compliance with treatment (Yes) (n = 275)**	88.4%	90.4%	86.3%	1.497 (0.708; 3.167)

‡ Mean (standard deviation). CVR: Cardiovascular risk. Mini-CHAL: quality of life questionnaire validated for use with patients with high blood pressure (Zero [best health level] to 30 [worst health level]) for mental status and 0 to 18 for Somatic status

## Discussion

### Main findings of the study

A large number of patients in each arm achieved good control of their HBP by 12 months; before adjusting for any influencing variables, no significant differences were seen between the arms. However, after adjusting for age, baseline cholesterol level, and baseline BP, the subjects in the Educore arm were more likely to have achieved good control of their HBP at this time (OR 1.57; 95%CI (1.02–2.41); ITT analysis). The good results obtained in both arms might be explained by patient motivation, the interest shown by the health professionals in improving their clinical practice, or because of the Hawthorne effect, i.e., the feeling of being observed spurring the medical professionals and/or patients to make greater efforts.

No significant differences were seen at any time point between the Educore and usual care arms in terms of any secondary outcome variable.

### Strengths and limitations

According to the indications of the PRECIS tool [24], the design of the study was pragmatic. This is further highlighted in that the results were of importance to the patients involved. The patients all had poorly controlled HBP, and all were recruited at primary healthcare centres where all were treated. The present intervention was independent of the therapeutic regimen prescribed, and in purely clinical terms the prescribing doctors in all cases followed the usual practice for the care of such patients. It is normal for patients with HBP to come for 6-monthly check-ups—more often if control remains poor (and allowed under the present trial conditions). The results for the main outcome variable—good control of HBP at 12 months—were subjected to per protocol and ITT analysis.

Before the study started, the medical professionals assigned to the Educore arm attended a training session during which they were given instructions to facilitate the passing of information on CVR to their patients at their baseline visit. However, in order for the follow-up period to be as similar as possible to everyday practice, it was left up to them to decide whether to provide reinforcement at the 6 and 12 month visits. The results obtained therefore reflect those that might be obtained in real clinical practice.

The study suffers from several limitations. First, since the trial could not be performed blind, there may have been contamination between patients [23]. For this reason a cluster study design was chosen. Second, the health centres were not chosen randomly but for the sake of convenience; all had previously been involved in research and/or were teaching centres. This, it was believed, would help ensure the motivation of their staff (who were not compensated economically in any way), help ensure adequate recruitment, and minimise losses to follow-up. Third, there may have been some bias in the selection of the patients; because -the patients were recruited after the health professionals were informed of their centre assignment. However, since an improvement was seen in both arms, this potential bias seems not to have had much effect. Fourth, difficulties arose during the study recruitment. Most of patients with HBP who presented poor control had diabetes mellitus or CVD (exclusion criteria). At the beginning, to promote the involvement of the professionals and to achieve an adequate recruitment, the health centres were selected for convenience and the professionals were volunteers. However, a significant percentage of professionals abandoned their participation in the study for reasons unrelated to the development of the study (changes of professionals between centres). This affected several of the participating professionals who could not be replaced in that phase of the study.

Fifth, in line with the pragmatic nature of the study, BP measurements were obtained as part of normal practice. The results obtained were therefore open to operator variation, i.e., between the people taking the measurements. Similarly, the doctors who participated recruiting patients were also those who treated them and who applied the intervention protocol. Sixth, it must be assumed that some heterogeneity existed between the participating physicians with respect to the time spent by each in delivering the information.

Finally, only 70% of the patients finished the study. Given the assumption of a 10% loss to follow-up, the final sample was smaller than that hoped for, reducing the power of the conclusions that can be drawn. It has been suggested [25], however, that with 30% 'missing completely at random' data, any estimation for an effect is very similar to that which would be obtained if all the data were available. In the present work, the differences between the results for the per protocol and ITT analyses were very small.

### Comparison with other studies

In a trial involving non-pharmacological interventions aimed at improving the control of HBP in patients in whom it was uncontrolled [26], improvements were also seen in both the intervention and control arms, with no significant differences between them. However, unlike that seen in the present work, the percentage control achieved in this earlier trial did not surpass 50% in either arm.

In another study [27], the control of HBP at 12 months of follow-up in patients with diabetes and HBP reached 22% in the group structured intervention arm, and 12% in the control arm. The improvement in SBP and DBP was slightly lower than that seen in the present study in both arms. Other authors [7] have reported a difference of 20.6% in the increase of patients achieving control over their HBP (multicomponent intervention compared with usual care).

The challenge in communicating risk to patients is to make the information provided understandable, usable and relevant [28,29]. It has been suggested [30] that one should exercise caution when providing information since the capacity of different people to absorb it will naturally vary. It may be a good idea to try to provide comparisons that can facilitate comprehension, for example by comparing the risk of a car crash to the risk of suffering a cerebrovascular accident in patients with atrial fibrillation. One study performed in the primary setting [10] reported that the majority (73%) of family doctors communicated CVR to their patients verbally, but it was found that patients understood this information better if it was presented in visual and numerical formats. Other authors [31] report that patients who receive written information (e.g., pamphlets) achieve a greater level of understanding than those who do not receive such-formatted information, and this information can be more effective if illustrations and graphs are used. Still others [32] indicate that the comprehension of risk by patients is rarely checked, leading to a lack of adherence to the recommendations given.

### Implications of the study findings

In conclusion, both usual care and the Educore intervention improved the percentage of patients with good control of their HBP at 12 months. However, after adjusting for age, baseline cholesterol level, and baseline BP, the subjects in the Educore arm were more likely to have achieved good control of their HBP at this time. It should be remembered, in this context, that the risk associated with an increase in BP is continuous; a 2 mmHg increase in SBP is associated with a 7% increase in the risk of mortality by myocardial infarction, and a 10% increase in the risk of mortality by cerebrovascular accident [5]. In the present work, a mean reduction in SBP of 16 mmHg was seen in the Educore intervention arm compared to 15 mmHg in the usual care arm.

Moreover, the differences observed between groups were small because, as we said, the design of the study was pragmatic. The Spanish National Health System could be another reason. The usual care in primary healthcare for patients with HBP is protocolized and includes standardized information for the patient that could be sufficient. Possibly in subgroups of patients with worse BP control, the Educore intervention could provide greater benefit.

The Educore intervention can easily be integrated into usual practice, and has no associated risks. It is possible that the smaller-than-expected size of the difference observed between the present trial arms was due to the doctors in the usual care arm modifying their practice (perhaps the consequence of their knowing they were involved in a trial). However, the results suggest that providing patients with visual information about CVR, for example by revealing them their SCORE rating, facilitates their achieving control over their HBP. More research might determine whether reinforcement of the intervention message can improve results further.

## Supporting information

S1 File CONSORT checklist (cluster randomized trial). (PDF)

S2 File Study protocol. (PDF)
