# Supplementary material for: A primary healthcare information intervention for communicating cardiovascular risk to patients with poorly controlled hypertension: The Education and Coronary Risk Evaluation (Educore) study—A pragmatic, cluster-randomized trial
Source: PLoS One. 2020 Jan 23;15(1):e0226398. doi: 10.1371/journal.pone.0226398 (PMC6977759; doi:10.1371/journal.pone.0226398)
Supplement: S2 File — (PDF) [file pone.0226398.s002.pdf]

Rodríguez-Salceda et al. BMC Public Health 2010, 10:449
http://www.biomedcentral.com/1471-2458/10/449

BMC Public Health

STUDY PROTOCOL Open Access

EDUCORE project: a clinical trial, randomised by clusters, to assess the effect of a visual learning method on blood pressure control in the primary healthcare setting

Isidro Rodríguez-Salceda[1], Esperanza Escortell-Mayor[2*], Milagros Rico-Blázquez[3], Rosario Riesgo-Fuertes[4], Angel Asúnsolo-del Barco[5], Antonio Valdivia-Pérez[6], Isabel del Cura-González[7], Ana B García-Cañón[8], María F Ortiz-Jiménez[9], Luisa Cabello-Ballesteros[10], Sofia Garrido-Elustondo[11], Laura Chamorro-González[12], Ricardo Rodríguez-Barrientos[13], EDUCORE Group[14]

Abstract

Background: High blood pressure (HBP) is a major risk factor for cardiovascular disease (CVD). European hypertension and cardiology societies as well as expert committees on CVD prevention recommend stratifying cardiovascular risk using the SCORE method, the modification of lifestyles to prevent CVD, and achieving good control over risk factors. The EDUCORE (Education and Coronary Risk Evaluation) project aims to determine whether the use of a cardiovascular risk visual learning method - the EDUCORE method - is more effective than normal clinical practice in improving the control of blood pressure within one year in patients with poorly controlled hypertension but no background of CVD;

Methods/Design: This work describes a protocol for a clinical trial, randomised by clusters and involving 22 primary healthcare clinics, to test the effectiveness of the EDUCORE method. The number of patients required was 736, all between 40 and 65 years of age (n = 368 in the EDUCORE and control groups), all of whom had been diagnosed with HBP at least one year ago, and all of whom had poorly controlled hypertension (systolic blood pressure ≥ 140 mmHg and/or diastolic ≥ 90 mmHg). All personnel taking part were explained the trial and trained in its methodology. The EDUCORE method contemplates the visualisation of low risk SCORE scores using images embodying different stages of a high risk action, plus the receipt of a pamphlet explaining how to better maintain cardiac health. The main outcome variable was the control of blood pressure; secondary outcome variables included the SCORE score, therapeutic compliance, quality of life, and total cholesterol level. All outcome variables were measured at the beginning of the experimental period and again at 6 and 12 months. Information on sex, age, educational level, physical activity, body mass index, consumption of medications, change of treatment and blood analysis results was also recorded;

Discussion: The EDUCORE method could provide a simple, inexpensive means of improving blood pressure control, and perhaps other health problems, in the primary healthcare setting;

Trial registration: The trial was registered with ClinicalTrials.gov, number NCT01155973 [http://ClinicalTrials.gov].

* Correspondence: eescortell.gapm03@salud.madrid.org
[2]Gerencia Atención Primaria Área 3, Servicio Madrileño de Salud, Alcalá de Henares (Madrid), Spain

© 2010 Rodríguez-Salceda et al; licensee BioMed Central Ltd. This is an Open Access article distributed under the terms of the Creative Commons Attribution License (http://creativecommons.org/licenses/by/2.0), which permits unrestricted use, distribution, and reproduction in any medium, provided the original work is properly cited.

Background

High blood pressure and cardiovascular disease

In the year 2000, the worldwide prevalence of high blood pressure (HBP) among the adult population was 26.4% [1]. The highest prevalence figures are seen in the most developed regions - about 44% in Europe and 28% in the USA [2]. In Spain the figure is around 35%, reaching as high as 60% in people over 60 years of age [3]. This high prevalence is associated with increased rates of premature mortality and incapacity. Hypertension was responsible for 7.6 million early deaths in 2001 (13.5% of total mortality), along with 92 million cases of incapacity (6% of the total), and it is thought to be the cause of 54% of all strokes and 47% of all cases of ischaemic heart disease [4]. In Spain, over 30.0% of all deaths are owed to HBP [3]. The prospects for the future are even worse; it is believed HBP figures for the year 2025 will show a 60% increase over those of 2000 [1]. An ageing population, improvements in treatment and a growing epidemic of obesity underlie this expected increase [1,3,5].

Control of HBP

The control of HBP in Europe is far from optimum [6-8], and in southern Europe the proportion of hypertensive subjects who are under treatment and being monitored is smaller than in the continent's north [9]. The CONTROLPRES studies, undertaken in Spain in the primary healthcare setting, have, however, shown some improvement; between 1990 and 2003 the number of people in whom good control of HBP was achieved increased by 25% [10]. This amelioration was confirmed in the PRESCAP 2006 study [11], in which control was achieved in 40%, rising to 44% in the recently published (2009) MADRIC study [12]. However, the figures clearly show there is room for further improvement. Among the causes of poor control of HBP are late detection, the late start of treatment, poor treatment compliance (a common problem in patients with chronic conditions) [13] and clinical or therapeutic inertia [13-15].

Calculating cardiovascular risk as a clinical and preventive strategy

In recent years, many studies have investigated the use of tables based on the Framingham study [16-19] (e.g., the Anderson, Wilson, DORICA or REGICOR tables) for calculating cardiovascular risk (CVR). However, since 2003 the tables produced by the SCORE project have been recommended for use in Europe. These are derived from data harvested from 12 cohorts of patients, one of which is Spanish [20]. Although the calibration of the SCORE table for use in Spain was published in 2007 [21], the fourth European Cardiovascular Disease Prevention in Clinical Practice Guidelines [22], along with their adaptation by the Spanish Interdisciplinary Committee for Cardiovascular Prevention (*Comité Español Interdisciplinario para la Prevención Cardiovascular*: CEIPC) [23], recommend the use of the low risk SCORE table [22,23]. The healthcare instructions issued by the Madrid Region (*Comunidad de Madrid;* Revision 2009) [24] recommend the same [available at: http://www.madrid.org].

In randomised clinical trials, several strategies aimed at health professionals and patients have been shown capable of improving compliance with antihypertension therapy [25] and of reducing therapeutic inertia [26]. The best would seem to target both [27]. These have led to documented improvements in blood pressure [28,29], changes in lifestyle [30], reduced coronary risk and reduced overall cardiovascular mortality [31].

As a strategy, improving the perception of risk is closely related to making behavioural changes that affect health [32]. Leal et al. [33] report how an intervention that showed patients their CVR, assessed using the Framingham model, reduced this risk within one year. This was particularly true for patients at high or very high risk. In addition, in the IMPALA study, which examined the effectiveness of a nursing intervention on the perception of CVR calculated using the SCORE method, CVR fell significantly in the intervention group and, in fact, in the control group [34,35].

Ways of communicating CVR to patients

It is unclear which communication format (graphs, tables, percentages or rates) is the best to use in educational interventions, although each type has its own advantages [36]. Some authors suggest visual formats may be the best way to describe the risks and benefits of treatment [37] since effectiveness is not conditioned by the mathematical knowledge or general educational level of the patient [38]. Probabilistic information offered via a pictogram was found to be particularly useful with patients of low educational level when assessing the risks and benefits of pharmacological treatment [39], and communication via icons and matrices has been reported more precise than the use of percentages, independent of recipient educational level or age [40].

Affective communication can improve the perception of risk in high risk patients, and alleviate concerns in those whose risk is low [41]. The EDUCORE method (Education and Coronary Risk Evaluation) may provide a means of facilitating such communication. This method was developed following a proposal by one of the present authors and has been informally tested in patients at a primary healthcare centre over the course of a year.

Aim

The aim of the present work is to determine whether the EDUCORE method for communicating cardiovascular risk in patients with poorly controlled HBP, but no history of CVD, is more effective than normal practice in terms of improving the control of blood pressure at one year.

Methods/Design

Study characteristics

The present work involves a two-year community, parallel clinical trial, randomised by clusters. Twenty two primary healthcare centres (PHCC) in the Madrid Region (*Comunidad de Madrid*) took part. The recruitment of these centres was undertaken between March and May 2009. The research group was comprised of 166 persons: 148 general practitioners and nurses (clinical assistance group), and 19 scientific researchers (technical group). A member of the clinical assistance team at each participating centre was appointed as liaison officer between the centre and the technical group.

The study protocol was approved by the Area 3 Research Ethics Committee (*Comité de Ética de Investigación Clínica del Área 3*) (2009/24/06), and met all good clinical practice demands. The trial was registered with ClinicalTrials.gov, number NCT01155973 [http://ClinicalTrials.gov].

The actual patient recruitment started on June 1st, 2010.

Patients

1. *Inclusion criteria*. All patients were required to be:

a. Attending patients diagnosed with HBP (systolic blood pressure ≥ 140 mmHg and/or diastolic blood pressure ≥ 90 mmHg) at least one year ago, with analytical (complete biochemical, cholesterol, serum creatinine and microalbuminuria results) and ECG results available, whose blood pressure was poorly controlled (last determination systolic ≥ 140 mmHg and/or diastolic ≥ 90 mmHg), and for whom information on organ involvement was available.
b. Aged 40-65 years.
c. To be able to meet the demands of the trial, i.e., to have no intention of moving, to be localisable for the next year, and to have the capacity to understand the questionnaires presented.
d. To have given signed, informed consent to be included in the study, and to meet no exclusion criterion.

2. *Exclusion criteria*. Patients were required not to have suffered a major cardiovascular event (cerebrovascular accident, heart disease, kidney disease, peripheral artery disease, advanced retinopathy [grades III/IV]), or to be suffering diabetes mellitus, a serious chronic disease with a notable impact on physical or psychological status (immobility patients, multi-system disease, serious deterioration of quality of life owed to chronic disease), or to be unable to collaborate (patients with dementia or other psychopathologies).

Randomisation

Sampling was performed by clusters, the PHCCs being the randomisation units. This minimised possible contamination effects between centres. An independent statistician randomly assigned the 22 PHCCs (each of which were randomly assigned a code) to either the EDUCORE method group or a control (normal practice) group (11 centres in each group) using Epidat 3.1 software.

Consecutive patients were chosen to minimise the risk of bias in their selection. Figure 1 shows the process followed (Figure 1. Patients' recruitment). During consultations, patients were informed about the study and asked whether they would like to take part. Those who accepted were asked to give their signed consent, and checks were made to ensure they met all inclusion criteria but no exclusion criterion.

Sample size

Method of calculation. For an alpha of 0.05, a power of 80%, and in order to detect an increase in good control of blood pressure of 15% in the EDUCORE group, the overall sample size required was 354 patients (177 in each arm of the study). Since randomisation was by clusters, the sample size had to be larger than if simple randomisation had been performed, in order to take into account the design effect (DE). The DE was calculated as follows: $DE = 1 + (n_c - 1) * ICC$ (where n_c is the mean number of individuals in the cluster, and ICC the intracluster correlation coefficient) [42]. Bearing in mind that the ICC range for trials randomised by clusters involving systolic blood pressure is 0 - 0.052 [42], the ICC in the present work was deemed to be 0.03. The mean cluster size was assumed to be 30 patients. Given these assumptions, and expecting a 10% loss rate at one year, the final sample size required was 736 patients (368 in each arm).

Masking

In a study of this type it is impossible to mask the intervention. To mask the analysis of data, the persons charged with this task were blind to the arm to which each PHCC had been assigned.

The intervention

Before beginning the field work, all the healthcare professionals taking part attended a number of training

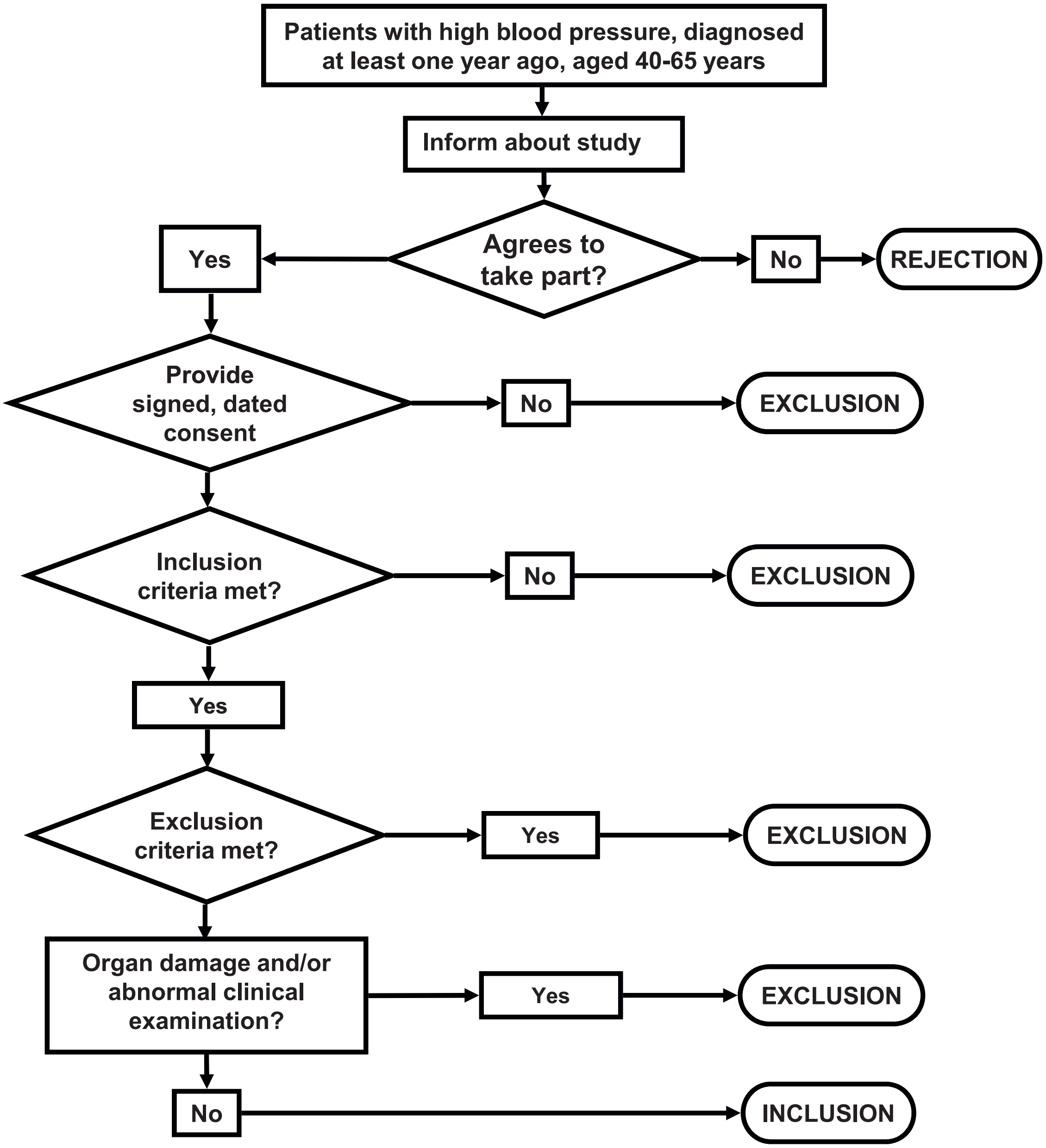

Figure 1 Patients' recruitment.

sessions. These involved a 'presentation day' on which members of the technical group explained the work to the liaison officers. This was followed by 2-3 h sessions aimed at the members of the clinical assistance teams at each of the participating centres. These sessions discussed the methodology of the study, the interventions (the EDUCORE method and normal practice), the work plan (organisation and data collection), the storage of data, and ethical, legal and authorship questions.

Table 1 summarise the steps followed at each appointment. Patients attended five appointments over the time of the study period. Each appointment lasted 10-30 min.

Normal practice group

The steps followed in the control arm included:

1. **Use of low risk SCORE table** [23]:

   a. Calculation of CVR at appointments 1, 3 and 5 (at the beginning of the experimental period and again at 6 and 12 months).

Table 1 Actions at each patient appointment

**Appointment 1**	
**Recruitment: may require an extra patient appointment to collect all the information necessary for a decision on inclusion/exclusion to be made, to inform the patient about the study, and to request signed consent to be included. See Figure 1.**	
**Actions performed in both study arms (GP/nurse)**	**Additional actions performed in the EDUCORE arm (GP)**
Collection of sociodemographic data: sex, age, educational level, physical activity, use of tobacco, consumption of medications, body mass index.	Use of EDUCORE method.
Taking of blood pressure. Completion of MiniCHAL and Morisky-Green questionnaires.	
Calculation of SCORE score and extrapolation to 60 years of age.	
Define interventions and therapeutic objectives for CVR, HBP, total cholesterol and tobacco use	
At the end of the appointment, inform patient of therapeutic plan (pharmacological/non-pharmacological). Record changes in drugs prescribed.	
**Appointment 2 (at 3 months)**	
**Actions in both arms (GP/nurse)**	**No additional actions in the EDUCORE arm**
Record blood pressure and degree of control.	
Review pharmacological treatment.	
**Appointment 3 (at 6 months)**	
**Actions performed in both study arms (GP/nurse)**	**Additional actions performed in the EDUCORE arm (GP)**
Record blood pressure and degree of control. Completion of Morisky-Green questionnaire.	Use of EDUCORE method.
Review pharmacological treatment.	
Calculation of SCORE score and extrapolation to 60 years of age.	
**Appointment 4 (at 9 months)**	
**Actions in both arms (GP/nurse)**	**No additional actions in the EDUCORE arm**
Record blood pressure and degree of control.	
Review pharmacological treatment.	
**Appointment 5 (at 12 months)**	
**Actions performed in both study arms (GP/nurse)**	**No additional actions in the EDUCORE arm**
Record blood pressure and degree of control.	
Completion of MiniCHAL and Morisky-Green questionnaires.	
Record physical activity and changes in number of packets of cigarettes smoked/year in smokers.	
Calculation of SCORE score and extrapolation to 60 years of age.	
Perform annual blood analysis. Review pharmacological treatment.	
Record changes in clinical variables (consumption of medications, body mass index).	

b. Calculation of the projected CVR at 60 years of age for all patients under this age.

2. **Verbally informing the patient of his/her CVR.** Neither the low risk SCORE table nor any other visual aid (images, graphs, tables) was shown to the patients until the end of the study (appointment 5).

3. **Giving advice/verbal information on risk factors**.

The criteria followed for the assessment and control of CVR were those published by the Madrid Regional Health Service (available at: [http://www.madrid.org/cs/Satellite?cid=1142584869941&language=es&pagename=PortalSalud%2FPage%2FPTSA_pintarContenidoFinal&vest=1142584869941] [24].

The EDUCORE intervention group

The steps followed in the EDUCORE arm included:

1. **Use of low risk SCORE table** [23]:

a. Calculation of CVR at appointments 1, 3 and 5 (at the beginning of the experimental period and again at 6 and 12 months).

b. Calculation of the projected CVR at 60 years of age for all patients under this age.

2. **Use of visual impact images**. Patients were shown images embodying different stages of a high risk action using the consultation room's computer with the aim of increasing consciousness regarding risk-entailing habits.

3. **Handing the patient a pamphlet**. This pamphlet contained advice on how to maintain cardiovascular health plus the low risk SCORE table with the patient's current score marked.

Variables

Outcome variables

The main outcome variable measured in both groups was the control of blood pressure (systolic blood pressure < 140 mmHg and dyastolic blood pressure < 90 mmHg). The blood pressure was determined using the Riva-Rocci method and listening for Korotkoff sounds, using validated and calibrated sphygmomanometers). The secondary outcome variables recorded were: CVR (measured using the low risk SCORE table), total cholesterol level, the use of tobacco, therapeutic compliance (measured using the Morisky-Green treatment compliance questionnaire) [43], and quality of life (measured using the miniCHAL quality of life questionnaire validated for use with patients with HBP) [44].

Sociodemographic variables recorded

These included sex, age and level of education (low, primary studies only; medium, high school completed, technical training or other non-university studies completed; high, university level education completed). Patient physical activity was measured using a questionnaire (recorded in metabolic equivalent hours per week) [45].

Clinical variables recorded

These included body mass index (weight in kg/height in cm^2), use of tobacco (current smoker, ex-smoker, never smoker; smokers were asked to declare the number of packets smoked per year), total cholesterol and LDL-cholesterol (mg/dl), serum creatinine (mg/dl) and micro-albuminuria/creatinine (mg/g). The consumption of cardiovascular medication (angiotensin converting enzyme inhibitors, angiotensin II receptor antagonists, diuretics, beta blockers, alpha blockers, renin direct inhibitors, and lipid lowering drugs) at the beginning and end of the study was also recorded. Changes in medication were also recorded, as were the reasons for such change, including secondary effects and ineffectiveness. The type of change made to antihypertension treatment was also recorded, including increasing/reducing dose, drug associations, suspensions and substitutions.

All data (excluding patient identification data) were recorded in an electronic database (previously validated in preliminary studies) searchable only by the EDUCORE group via a corporative intranet system. This centralised the collection of data, facilitating later analysis.

Variables recorded for health professionals

These included sex, age, professional category (general practitioner, nurse), the date of qualification, and the PHCC where services were rendered. This facilitated the description of the clinical assistance personnel associated with each cluster.

Patients who declined to participate

The number of patients who declined to participate was recorded, as required by the CONSORT statement [46].

Losses and withdrawals

The causes of all losses and withdrawals (voluntary withdrawal, change of residence, comorbidity or death, cause of death [cardiovascular or other]) were recorded. When patients failed to attend an appointment, two attempts were made to enter into contact by telephone.

Researcher communication

A web page was used to facilitate the monitoring of the work performed and to make the project more visible http://www.educore.es.

Analysis

The use of randomisation by clusters conditions the statistical analysis that can be employed, especially in the calculation of confidence intervals and the testing of hypotheses. The following analyses were undertaken:

1. **Descriptive analysis** and the description of the profile of patients who abandoned the study plus their reason for withdrawal.

2. **Baseline comparison** of the two intervention groups in terms of the analytical variables measured, prognostic factors and descriptive variables, using appropriate bivariate statistical tests.

3. **Effect of the EDUCORE method on blood pressure control (main outcome variable):** i.e., a comparison of the proportion of patients with good/bad control in the two study arms using the Chi-squared test, and the calculation of confidence intervals at 6 months and 1 year after patient inclusion. Logistic regression with random effects was used to adjust for prognostic factors; the dependent variable was good/bad control of patient blood pressure, and the independent variable the intervention group to which each patient belonged. Confounding factors or factors that might alter the effect recorded were taken into account in this analysis.

4. **Effect of the EDUCORE method on secondary outcome variables** (CVR, cholesterol levels, use of tobacco, therapeutic compliance and quality of life) using appropriate statistical tests.

All statistical tests were performed with intention to treat. The 'last observation carried forward' was used for missing data [47]. Significance was set at $P < 0.05$. All calculations were made using SPSS© v.18 software.

Discussion

Educational intervention strategies aimed at either patients or health professionals can provide benefits in the control of HBP [27,31,33]. The visual presentation of risk for different health problems, and using different formats, can improve the comprehension of risk and the motivation on the part of patients to change their lifestyles [32]. The EDUCORE method, which aims to improve the control of HBP by targeting both patients and health professionals, is simple, economic, and makes use of computer facilities already available in consultation rooms. If it proves to be effective, it might be used with certain other chronic diseases managed at PHCCs, perhaps providing further benefits to community health.

The study design used in this work is, however, subject to certain limitations. Firstly, the intervention cannot be masked, which could influence the assessment of its effect. Certainly, the possibility of contamination exists if the patients involved communicate with one another [46]. For this reason randomisation by clusters was employed. It was assumed that the number of clusters was sufficient for the random assignment of PHCCs to one arm or the other to compensate for such potential confounding factors.

In practice, the percentage of patients with good control of their HBP that can be reached, though improvable, is limited. Thus, achieving a very large impact with the EDUCORE method might be difficult. For this very reason, any positive effect achieved - even though small in magnitude - could be understood to represent success, especially in this disease of such great pubic health importance.

The conclusions drawn from this study might influence clinical practice and research into blood pressure control. In the clinical setting, the reduction of CVR in patients with HBP could have an important effect on health and quality of life.

Abbreviations

CVD: Cardiovascular disease; CVR: Cardiovascular risk; CEIPC: *Comité Español Interdisciplinario para la Prevención Cardiovascular*; DE: Design effect; EDUCORE: Education and Coronary Risk Evaluation; ECG: Electrocardiogram; GP: General practitioner; HBP: High blood pressure; ICC: Intracluster correlation coefficient; n_C: Number of individuals in a cluster; SCORE: Systematic Coronary Risk Evaluation;

Acknowledgements

The EDUCORE group

Clinical assistance group:

Primary healthcare centre (PHCC) Alcalá de Guadaira (Madrid): Ángela Lorenzo Lobato (*Liason Officer*), Ángeles I Tolosa Monton, Carmen Latorre de la Cruz, Josefa Gutiérrez Cabrera, Silvia Colinas Alonso, Diego José Villalvilla Soria.

PHCC Pavones (Madrid): Mercedes Ibáñez Brillas (*Liason Officer*), Inmaculada Hernández Beltrán, María Huertas Uhagon, Juan Ramón Iglesias Quintana.

PHCC Artilleros (Madrid): Riansares López Palomar (*Liason Officer*), Mª Begoña Ayuso de la Torre.

PHCC La Veredilla (Torrejón de Ardoz): Isidro Rodríguez Salceda (*Liason Officer*), Ana M Gómez Calvo, Carmen Alonso Villagra, Carmen Metola Serrano, Elena Rodríguez Quiroga, Rosa Blanca Pérez García, Raúl Ramírez Gutiérrez, Mercedes Fernández Ortega, Roberto Carrascoso Calvo, Inés Olga Bermejo Mayoral, Alicia Jorge Formariz, Luisa Lavilla Arnal, Carmen Yáñez Campos, Milagros Juaristi Lascurain, Antonia Torrenova.

PHCC Manuel Merino (Alcalá de Henares): Gloria de la Sierra Ocaña (*Liason Officer*), Mercedes Araujo Calvo, Jorge Geanini Torres, Elena Yubero Esteban, Angustias Atienza Panadero, Jesús Miguel Rodríguez Collada.

PHCC Fresnos (Torrejón de Ardoz): Ana Herrero Fuentes (*Liason Officer*), Encarnación Tornay Muñoz, Encarnación Serrano Serrano, Julia Torres Morales.

PHCC Mª Guzmán (Alcalá de Henares): José M Martín Moros (*Liason Officer*), Luisa Patón Cueva.

PHCC Fronteras (Torrejón de Ardoz): Alberto de Miguel Ballano (*Liason Officer*), Isabel Peláez Parra, Aurora Maestro Martín, Montserrat Sanz Herrero, Ana Muñoz Cildoz, Magdalena Sánchez Cabañas, Ana Belén Martínez Rubio, Pedro P Sánchez de la Calle, Inmaculada García García, Tamara Rodríguez Peral.

PHCC Mar Báltico (Madrid): Carlos Aguilera Collado (*Liason Officer*), Marta Maestre Casadomet, Juan Camarero Palacios, Isabel García del Río, José Carlos Villalba González, José Hernández Yela, Esperanza Villar Coloma, Isabel Agudo González, Ainhoa Camarero Miguel, Carmen Calvo Torres.

PHCC Chopera (Madrid): Juan C Obaya Rebollar (*Liason Officer*), Montserrat Barrios Encinas.

PHCC Águilas (Madrid): Marta Sanz Sanz (*Liason Officer*), Susana Rodríguez De Cos, Francisco García Ruiz, Gloria Acosta Vargas.

PHCC Andrés Mellado (Madrid): Juan Carlos Recio Velasco (*Liason Officer*), Laura Martín Arribas, Beatriz Álvarez Álvarez, Rosa M Pérez Dorado.

PHCC Maqueda (Madrid): Esther Arrojo Arias (*Liason Officer*), Esther Blázquez Hernández. Nieves Barboso León, Teresa Alzugaray García-Diego, Cristina Mora Casado, Ana Campillo Palomo, Amparo Suárez García, Mercedes Torrego Mateos.

PHCC Parque Loranca (Fuenlabrada): Paz Vítores Picón (*Liason Officer*), Marisa Herrera García, María J Rojas, Marta Núñez, Mª Victoria Rollon, Asunción San Juan, Ana Cifuentes, Sara Casero, Susana Hernández, Luisa Quilchano, Luis Pastor, Gema Téllez, Arturo Rodríguez, Ángeles Vilches.

PHCC Panaderas (Fuenlabrada): Teresa Gordillo de la Cruz (*Liason Officer*), Cristina Ramos Díaz, María Alonso Ovies, Josefa Fernández García.

PHCC Francia (Fuenlabrada): Ernesto Cerrada Cerrada (*Liason Officer*), Eugenia Hernaiz, Andrés Tellez, Rafael Camps, Marisa Valles, Mª Carmen Morales.

PHCC Santa Isabel (Leganés): Pedro Herrera (*Liason Officer*), Jesús García Soltero, Josefina Galán Esteban, Nazareth González Sánchez, Antonio Redondo Horcajo, Elisabeth Nieto Villalón, Francisco Alba, Carlos Vicente Sánchez, Rosa Fernández García, Ángel Delgado Delgado, Rosa M Villena Romero, Sandra García Casasola.

PHCC M Jesus Hereza (Leganés): María J Bedoya (*Liason Officer*), Raquel Vignolo Román, Luisa Illescas Sánchez, Orquídea Sales Aguade, Ángeles Rollán Hernández, Teresa Fontova Cemelí, Maite Rollán Landeras, Juanjo Cercas Gil.

PHCC Mª Ángeles López Gómez (Leganés): Julio Sánchez Salvador (*Liason Officer*), Luz D Torres Romo, Beatriz López Serrano, Arancha Martín Ramos.

PHCC Mendiguchía (Leganés): Eduardo Díaz García (*Liason Officer*), Gema Vázquez Salado, Mar Rubio Moreno, Belinda Pinel Vaquerizo, Gerardo García Sánchez, Marta Álvarez de la Riva.

PHCC Pinto (Pinto): Beatriz Herrera Sánchez (*Liason Officer*), José Antonio Tena Martínez, Belén Ortega Trompeta, Sagrario Campo Álvarez, Nélida Blanco Villafañe, Antonio de Torre López, Antonio Ruiz García, Teresa Sánchez López, Rosario Rico Pérez, José M Mansilla Domínguez.

PHCC Sector III (Getafe): Dolores de La Fuente Arriaran (*Liason Officer*), Ana Pavón Conde, Carmen Durán Luceño, Carmen Arnés Pérez, Josefa Vázquez

Gallego, Alicia Marqués Rebollo, Carmelina Sanz Velasco, Encarnación González Ortega.
Technical support group:
Teresa Sanz Cuesta. Unidad de Apoyo a la Investigación Clínica y Ensayos Clínicos en Atención Primaria (CAIBER), Área de Investigación, Agencia Laín Entralgo (ALE), Consejería de Sanidad, Comunidad de Madrid, Madrid, Spain.
Susana Monge Corella, Instituto de Salud Carlos III, Madrid, Spain.
Rocío González González, Unidad de Apoyo a la Investigación Clínica y Ensayos Clínicos en Atención Primaria (CAIBER), Área de Investigación, ALE, Consejería de Sanidad, Comunidad de Madrid, Madrid, Spain.
Miguel Ángel Salinero Fort, Subdirección de Investigación, Hospital Carlos III, Madrid, Spain.
Funding
This study was funded by the Spanish Ministry of Science and Innovation via the Instituto de Salud Carlos III, Subprograma de Proyectos de Investigación en Evaluación de Tecnologías Sanitarias y Servicios de Salud (09/90354).
The authors thank the following persons for their contributions to this work: Marta García-Solano, Dolores Otero-Criado, Rosa Zurdo-Baz and Raisa González-Pérez.

Author details

[1]Centro de Salud de Veredillas, Atención Primaria Área 3, Servicio Madrileño de Salud, Torrejón de Ardoz (Madrid), Spain. [2]Gerencia Atención Primaria Área 3, Servicio Madrileño de Salud, Alcalá de Henares (Madrid), Spain. [3]Dirección General de Atención Primaria de Madrid, Servicio Madrileño de Salud, Madrid, Spain. [4]Gerencia Atención Primaria Área 1, Servicio Madrileño de Salud, Madrid, Spain. [5]Universidad de Alcalá, Alcalá de Henares (Madrid), Spain. [6]Unidad de Medicina Preventiva, Hospital de Denia, Marina Salud, Agéncia Valenciana de Salut, Denia (Alicante), Spain. [7]Gerencia Atención Primaria Área 9, Servicio Madrileño de Salud, Leganés (Madrid), Spain. Facultad de Ciencias de la Salud, Universidad Rey Juan Carlos, Madrid, Spain. [8]Unidad de Apoyo a la Investigación Clínica y Ensayos Clínicos en Atención Primaria (CAIBER), Área de Investigación, Agencia Laín Entralgo, Consejería de Sanidad, Comunidad de Madrid, Madrid, Spain. [9]Centro de Salud Puerta de Madrid, Atención Primaria Área 3, Servicio Madrileño de Salud, Alcalá de Henares, Madrid, Spain. [10]Gerencia Atención Primaria Área 10, Servicio Madrileño de Salud, Getafe (Madrid), Spain. [11]Gerencia Atención Primaria Área 7, Servicio Madrileño de Salud, Madrid, Spain. [12]Hospital Universitario de Getafe, Servicio Madrileño de Salud, Getafe (Madrid), Spain. [13]Gerencia Atención Primaria Área 5, Servicio Madrileño de Salud, Madrid, Spain. [14]Servicio Madrileño de Salud (Atención Primaria), Comunidad de Madrid, Madrid, Spain.

Authors' contributions

IRS conceived of the study and participated in its design. EEM participated in the design and coordination of the study. IRS and EEM carried out the EDUCORE method. MRB, RRF, AAS, ICG, LCB, SGE and RRB (senior authors) participated in the design of the study. EEM, IRS, MRB, RRF and AAS directed the writing of the manuscript. Contributions were made by the remaining authors. All authors read and approved the final manuscript.

Competing interests

The authors declare that they have no competing interests.

Received: 13 July 2010 Accepted: 30 July 2010 Published: 30 July 2010

References

1. Kearney PM, Whelton M, Reynolds K, Muntner P, Whelton PK, He J: **Global burden of hypertension: analysis of worldwide data.** *Lancet* 2005, **365**:217-223.
2. Wolf-Maier K, Cooper RS, Banegas JR, Giampaoli S, Hense HW, Joffres M, *et al*: **Hypertension prevalence and blood pressure levels in 6 European countries, Canada, and the United States.** *JAMA* 2003, **289**:2363-2369.
3. Banegas-Banegas J: **Epidemiología de la hipertensión arterial en España. Situación actual y perspectivas.** *Hipertensión* 2005, **22**:353-362.
4. Lawes CM, Vander HS, Rodgers A: **Global burden of blood-pressure-related disease, 2001.** *Lancet* 2008, **371**:1513-1518.
5. Lloyd-Jones DM, Evans JC, Levy D: **Hypertension in adults across the age spectrum: current outcomes and control in the community.** *JAMA* 2005, **294**:466-472.
6. Wolf-Maier K, Cooper RS, Banegas JR, Giampaoli S, Hense HW, Joffres M, *et al*: **Hypertension prevalence and blood pressure levels in 6 European countries, Canada, and the United States.** *JAMA* 2003, **289**:2363-2369.
7. Wolf-Maier K, Cooper RS, Kramer H, Banegas JR, Giampaoli S, Joffres MR, *et al*: **Hypertension treatment and control in five European countries, Canada, and the United States.** *Hypertension* 2004, **43**:10-17.
8. Costanzo S, Di CA, Zito F, Krogh V, Siani A, Arnout J, *et al*: **Prevalence, awareness, treatment and control of hypertension in healthy unrelated male-female pairs of European regions: the dietary habit profile in European communities with different risk of myocardial infarction–the impact of migration as a model of gene-environment interaction project.** *J Hypertens* 2008, **26**:2303-2311.
9. Pereira M, Lunet N, Azevedo A, Barros H: **Differences in prevalence, awareness, treatment and control of hypertension between developing and developed countries.** *J Hypertens* 2009, **27**:963-975.
10. Coca A: **Evolución del control de la hipertensión arterial en Atención Primaria en España. Resultados del estudio Controlpres 2003.** *Hipertensión* 2005, **22**:5-14.
11. Llisterri Caro JL, Rodriguez Roca GC, onso Moreno FJ, Banegas B Jr, Gonzalez-Segura AD, Lou AS, *et al*: **Control de la presión arterial en la población hipertensa Española atendida en Atención Primaria. Estudio PRESCAP 2006.** *Med Clin (Barc)* 2008, **130**:681-687.
12. Castell MV, Martinez MA, Sanz J, Garcia PJ, en representación del grupo MAPA-Madrid: **Prevalence, awareness and control of arterial hypertension in a Spanish population. The MADRIC study.** *Med Clin (Barc)* 2009.
13. Heisler M, Hogan MM, Hofer TP, Schmittdiel JA, Pladevall M, Kerr EA: **When more is not better: treatment intensification among hypertensive patients with poor medication adherence.** *Circulation* 2008, **117**:2884-2892.
14. Ho PM, Bryson CL, Rumsfeld JS: **Medication adherence: its importance in cardiovascular outcomes.** *Circulation* 2009, **119**:3028-3035.
15. Márquez-Contreras E, Coca A, de la Figuera-von Wichmann , Divison JA, Llisterri JL, Sobrino J, *et al*: **Perfil de riesgo cardiovascular de los pacientes con hipertensión arterial no controlada. Estudio Control-Project.** *Med Clin (Barc)* 2007, **128**:86-91.
16. Aranceta J, Perez RC, Foz SM, Mantilla T, Serra ML, Moreno B, *et al*: **Tablas de evaluación del riesgo coronario adaptadas a la población Española. Estudio DORICA.** *Med Clin (Barc)* 2004, **123**:686-691.
17. Baena-Diez JM, Grau M, Sanchez-Perez R, tes-Vaques E, Salas-Gaetjens LH, Hernandez-Ibanez MR: **The REGICOR-calibrated function provides a better classification of high-risk patients on statin treatment in the Spanish population than the Framingham or SCORE classifications.** *Rev Esp Cardiol* 2009, **62**:1134-1140.
18. Cristóbal J, Lago F, de la Fuente J, González-Juanatey JR, Vázquez-Bellés P, Vila M: **Ecuación de Framinghan de Wilson y ecuación de REGICOR. Estudio comparativo.** *Rev Esp Cardiol* 2005, **58**:910-915.
19. de Cabrera LA, Rodriguez-Perez MC, del Castillo-Rodriguez JC, Brito-Diaz B, Perez-Mendez LI, de Muros FM, *et al*: **[Coronary risk in the population of the Canary Islands, Spain, using the Framingham function].** *Med Clin (Barc)* 2006, **126**:521-526.
20. Conroy RM, Pyorala K, Fitzgerald AP, Sans S, Menotti A, De BG, *et al*: **Estimation of ten-year risk of fatal cardiovascular disease in Europe: the SCORE project.** *Eur Heart J* 2003, **24**:987-1003.
21. Sans S, Fitzgerald AP, Royo D, Conroy R, Graham I: **Calibración de la tabla SCORE de riesgo cardiovascular para España.** *Rev Esp Cardiol* 2007, **60**:476-485.
22. Graham I, Atar D, Borch-Johnsen K, Boysen G, Burell G, Cifkova R, *et al*: **European guidelines on cardiovascular disease prevention in clinical practice: full text. Fourth Joint Task Force of the European Society of Cardiology and other societies on cardiovascular disease prevention in clinical practice (constituted by representatives of nine societies and by invited experts).** *Eur J Cardiovasc Prev Rehabil* 2007, **14(Suppl 2)**:S1-113.
23. Lobos JM, Royo-Bordonada MA, Brotons C, Álvarez-Sala L, Armario P, Maiques A: **Guía Europea de Prevención Cardiovascular en la práctica clínica. Adaptación Española del CEIPC 2008.** *Rev Esp Salud Pública* 2008, **82**:581-616.
24. **Servicio Madrileño de Salud. Cartera de Servicios estandarizados de Atención Primaria.** 2009 [http://www.madrid.org/cs/Satellite?cid=1142584869941&language=es&pagename=PortalSalud%2FPage%2FPTSA_pintarContenidoFinal&vest=1142584869941].

25. Márquez E, Martel N, Gil GV, Martín-De Pablos JL, De la Figuera-Von Wichman, Casado-Martínez JJ, *et al*: **Non-pharmacological intervention as a strategy to improve antihypertensive treatment compliance.** *Aten Primaria* 2009, **41**:501-510.
26. Márquez E, Martel N, Gil GV, Martín-De Pablos JL, De la Figuera-Von Wichman, Casado-Martínez JJ, *et al*: **Control of therapeutic inertia in the treatment of arterial hypertension by using different strategies.** *Aten Primaria* 2009, **41**:315-323.
27. Roumie CL, Elasy TA, Greevy R, Griffin MR, Liu X, Stone WJ, *et al*: **Improving blood pressure control through provider education, provider alerts, and patient education: a cluster randomized trial.** *Ann Intern Med* 2006, **145**:165-175.
28. Gómez-Marcos MA, García-Ortiz L, González-Elena LJ, Ramos-Delgado E, González-García AM, Parra-Sánchez J: **Efectividad de una intervención de mejora de calidad en el control de la presión arterial en Atención Primaria.** *Rev Clin Esp* 2006, **206**:428-434.
29. Hennessy S, Leonard CE, Yang W, Kimmel SE, Townsend RR, Wasserstein AG, *et al*: **Effectiveness of a two-part educational intervention to improve hypertension control: a cluster-randomized trial.** *Pharmacotherapy* 2006, **26**:1342-1347.
30. Mattila R, Malvivaara A, Kastarinen M, Kivela SL, Nissinen A: **Effectiveness of multidisciplinary lifestyle intervention for hypertension: a randomised controlled trial.** *J Hum Hypertens* 2003, **17**:199-205.
31. Gómez-Marcos MA, García-Ortiz L, González-Elena LJ, Sánchez Rodríguez A: **Efectividad de una intervención de mejora de calidad en la reducción del riesgo coronario y del riesgo de mortalidad cardiovascular en pacientes hipertensos.** *Aten Primaria* 2006, **37**:498-503.
32. Brewer NT, Chapman GB, Gibbons FX, Gerrard M, McCaul KD, Weinstein ND: **Meta-analysis of the relationship between risk perception and health behavior: the example of vaccination.** *Health Psychol* 2007, **26**:136-145.
33. Leal-Hernández M, Abellán-Alemán J, Ríos-Cano EJ, Martínez-Crespo J, Sebastián-Vicente B, Vicente-Martínez R: **Información sobre el riesgo cardiovascular a hipertensos seguidos en atención primaria. ¿Mejora nuestra eficacia?** *Aten Primaria* 2006, **38**:102-106.
34. Koelewijn-van Loon MS, van der WT, Ronda G, van SB, Winkens B, Elwyn G, *et al*: **Improving lifestyle and risk perception through patient involvement in nurse-led cardiovascular risk management: a cluster-randomized controlled trial in primary care.** *Prev Med* 2010, **50**:35-44.
35. Koelewijn-van Loon MS, van der WT, van SB, Ronda G, Winkens B, Severens JL, *et al*: **Involving patients in cardiovascular risk management with nurse-led clinics: a cluster randomized controlled trial.** *CMAJ* 2009, **181**:E267-E274.
36. Timmermans DR, Ockhuysen-Vermey CF, Henneman L: **Presenting health risk information in different formats: the effect on participants' cognitive and emotional evaluation and decisions.** *Patient Educ Couns* 2008, **73**:443-447.
37. Goodyear-Smith F, Arroll B, Chan L, Jackson R, Wells S, Kenealy T: **Patients prefer pictures to numbers to express cardiovascular benefit from treatment.** *Ann Fam Med* 2008, **6**:213-217.
38. Keller C, Siegrist M: **Effect of risk communication formats on risk perception depending on numeracy.** *Med Decis Making* 2009, **29**:483-490.
39. Hawley ST, Zikmund-Fisher B, Ubel P, Jancovic A, Lucas T, Fagerlin A: **The impact of the format of graphical presentation on health-related knowledge and treatment choices.** *Patient Educ Couns* 2008, **73**:448-455.
40. Galesic M, Garcia-Retamero R, Gigerenzer G: **Using icon arrays to communicate medical risks: overcoming low numeracy.** *Health Psychol* 2009, **28**:210-216.
41. Ancker JS, Chan C, Kukafka R: **Interactive graphics for expressing health risks: development and qualitative evaluation.** *J Health Commun* 2009, **14**:461-475.
42. Adams G, Gulliford MC, Ukoumunne OC, Eldridge S, Chinn S, Campbell MJ: **Patterns of intra-cluster correlation from primary care research to inform study design and analysis.** *J Clin Epidemiol* 2004, **57**:785-794.
43. Nogués-Solán X, Sorli-Redó ML, Villar-García J: **Instrumentos de medida de adherencia al tratamiento.** *An Med Interna* 2007, **24**:138-141.
44. Roca-Cusachs A, Badia X, Dalfó A, Gascón G, Abellán J, Lahoz R, *et al*: **Relación entre variables clínicas y terapéuticas y calidad de vida relacionada con la salud en pacientes con hipertensión arterial. Estudio MINICHAL.** *Med Clin (Barc)* 2003, **121**:12-17.
45. Meseguer CM, Galan I, Herruzo R, Zorrilla B, Rodríguez-Artalejo F: **Leisure-time physical activity in a southern European mediterranean country: adherence to recommendations and determining factors.** *Rev Esp Cardiol* 2009, **62**:1125-1133.
46. Campbell MK, Elbourne DR, Altman DG: **CONSORT statement: extension to cluster randomised trials.** *BMJ* 2004, **328**:702-708.
47. Salim A, Mackinnon A, Christensen H, Griffiths K: **Comparison of data analysis strategies for intent-to-treat analysis in pre-test-post-test designs with substantial dropout rates.** *Psychiatry Res* 2008, **160**:335-345.

Pre-publication history
The pre-publication history for this paper can be accessed here:
http://www.biomedcentral.com/1471-2458/10/449/prepub

doi:10.1186/1471-2458-10-449
Cite this article as: Rodríguez-Salceda *et al.*: EDUCORE project: a clinical trial, randomised by clusters, to assess the effect of a visual learning method on blood pressure control in the primary healthcare setting. *BMC Public Health* 2010 **10**:449.

Submit your next manuscript to BioMed Central and take full advantage of:

- Convenient online submission
- Thorough peer review
- No space constraints or color figure charges
- Immediate publication on acceptance
- Inclusion in PubMed, CAS, Scopus and Google Scholar
- Research which is freely available for redistribution

Submit your manuscript at
www.biomedcentral.com/submit

BioMed Central
